# SOCS-1 inhibition of type I interferon restrains *Staphylococcus aureus* skin host defense

**DOI:** 10.1371/journal.ppat.1009387

**Published:** 2021-03-10

**Authors:** Nathan Klopfenstein, Stephanie L. Brandt, Sydney Castellanos, Matthias Gunzer, Amondrea Blackman, C. Henrique Serezani

**Affiliations:** 1 Department of Medicine, Division of Infectious Diseases, Vanderbilt University Medical Center, Nashville, Tennessee, United States of America; 2 Department of Pathology, Microbiology, and Immunology, Vanderbilt University, Nashville, Tennessee, United States of America; 3 Vanderbilt Center for Immunobiology, Vanderbilt University Medical Center, Nashville, Tennessee, United States of America; 4 Vanderbilt Institute of Infection, Immunology and Inflammation, Vanderbilt University Medical Center, Nashville, Tennessee, United States of America; 5 Institute for Experimental Immunology and Imaging, University Hospital, University Duisburg-Essen, Hufelandstrasse Essen, Germany; 6 Leibniz-Institut für Analytische Wissenschaften—ISAS -e.V, Dortmund, Germany; Columbia University, UNITED STATES

## Abstract

The skin innate immune response to methicillin-resistant *Staphylococcus aureus* (MRSA) culminates in the formation of an abscess to prevent bacterial spread and tissue damage. Pathogen recognition receptors (PRRs) dictate the balance between microbial control and injury. Therefore, intracellular brakes are of fundamental importance to tune the appropriate host defense while inducing resolution. The intracellular inhibitor suppressor of cytokine signaling 1 (SOCS-1), a known JAK/STAT inhibitor, prevents the expression and actions of PRR adaptors and downstream effectors. Whether SOCS-1 is a molecular component of skin host defense remains to be determined. We hypothesized that SOCS-1 decreases type I interferon production and IFNAR-mediated antimicrobial effector functions, limiting the inflammatory response during skin infection. Our data show that MRSA skin infection enhances SOCS-1 expression, and both SOCS-1 inhibitor peptide-treated and myeloid-specific SOCS-1 deficient mice display decreased lesion size, bacterial loads, and increased abscess thickness when compared to wild-type mice treated with the scrambled peptide control. SOCS-1 deletion/inhibition increases phagocytosis and bacterial killing, dependent on nitric oxide release. SOCS-1 inhibition also increases the levels of type I and type II interferon levels *in vivo*. IFNAR deletion and antibody blockage abolished the beneficial effects of SOCS-1 inhibition *in vivo*. Notably, we unveiled that hyperglycemia triggers aberrant SOCS-1 expression that correlates with decreased overall IFN signatures in the infected skin. SOCS-1 inhibition restores skin host defense in the highly susceptible hyperglycemic mice. Overall, these data demonstrate a role for SOCS-1-mediated type I interferon actions in host defense and inflammation during MRSA skin infection.

## Author summary

The control of *Staphylococcus aureus* skin and soft tissue infections is dictated by the balance between pro- and anti-inflammatory actions in phagocytes and structural cells. Although most studies have focused on the actions of inflammatory mediators, the role of intracellular effectors that shape innate immune response during skin infection is not well understood. Here, we identified a heretofore unknown role of the intracellular negative regulator of the immune response suppressor of cytokine signaling 1 (SOCS-1) in the control of *S*. *aureus* skin infection. Myeloid-specific SOCS-1 expression is detrimental to skin infection by inhibiting type I interferon (IFN)-dependent nitric oxide production and bacterial killing. A SOCS-1 blocking peptide greatly increases skin host defense. Importantly, we linked increased SOCS-1 expression to impaired host defense in a model of hyperglycemia. Transcriptomic analysis showed overall decreased expression of IFN-related genes and genetic and pharmacological SOCS-1 blockage restored skin host defense in hyperglycemic mice. Together, our work demonstrates a previously undocumented detrimental role for SOCS1-dependent skin host defense and suggests that blocking SOCS-1 might enable the host to efficiently control infection in both homeostatic and hyperglycemic conditions.

## Introduction

*Staphylococcus aureus* is the leading cause of skin and soft tissue infections in the United States, accounting for almost 500,000 hospital admissions a year [1]. Although *S*. *aureus* colonizes ~30% of the population, it is well suited to breaching the skin barrier, resulting in localized or potentially more severe systemic infections. The resistance of *S*. *aureus* to multiple antibiotics, particularly methicillin-resistant *Staphylococcus aureus* (MRSA), has made treatment of these infections increasingly difficult [2]. With the rise in antimicrobial resistance, there is a compelling need for safe, inexpensive, and non-antibiotic approaches that do not directly attack the bacterial target (which can lead to resistance over time), but instead host-centered strategies to prevent and treat these bacterial infections.

Skin resident macrophages, along with recruited monocytes and neutrophils, are responsible for major events during *S*. *aureus* skin infection, including recognizing the infection, abscess formation, and resolution of the inflammatory response [3,4]. Macrophages orchestrate abscess formation by establishing the inflammatory tone, recruiting neutrophils, killing microbes, clearing dead cells, and initiating wound healing. Since the abscess harbors viable and necrotic neutrophils plus bacteria at its core, it must be tightly organized to prevent deeper infection and bacterial dissemination [4,5].

Phagocytes contribute to host defense during multiple stages of *S*. *aureus* skin infection. While skin resident macrophages are involved in the initial recognition and killing of the bacteria, these cells are primarily engaged in producing chemoattractants to promote neutrophil and monocyte recruitment to the skin. Recruited neutrophils are crucial in *S*. *aureus* elimination through phagocytosis and killing via the generation of antimicrobial peptides, reactive oxygen (ROS) and nitrogen (RNS) species, as well as neutrophil extracellular traps (NETs) within the abscess [5,6]. Neutrophil-derived IL-1β also plays a critical role in optimal neutrophil recruitment to the site of infection, proper abscess formation, and improved infection outcome [3,7]. Macrophages handle the resolution of the infection at the periphery of the abscess, which clears out the dead cell debris and breakdown the fibrous abscess capsule to allow for tissue healing and scar formation [4,8].

Various pathogen recognition receptors (PRRs) recognize *S*. *aureus*, including scavenger receptors and the Toll-like receptors (TLRs) 1,2, 6, and 9 [9]. Both TLRs and IL1R utilize the adaptor protein myeloid differentiation primary response 88 (MyD88) for intracellular signaling [7,10]. MyD88 is a critical component of the host immune response to *S*. *aureus* skin infections [5,11]. MyD88-deficient mice demonstrate impaired abscess formation and neutrophil recruitment during *S*. *aureus* skin, bone, and kidney infection, correlating with worse infection outcomes [3,7,10]. MyD88-dependent signaling culminates in activating different transcription factors such as NFκB, AP1, and IRFs [11]. Interestingly, TLR9 utilizes MyD88 to induce the production of type I interferons (IFNs). Furthermore, TLR9-mediated IFNβ production has been shown to influence bacterial pathogenicity during *S*. *aureus* infection in the lungs [12,13].

Both type I and type II interferons are well-known enhancers of antimicrobial effector function in phagocytes. IFNγ increases host defense against a variety of pathogens (virus, bacteria, fungi, and parasites) [8,14,15], and the role of type I IFNs in the control of viral infections is well established while their role in bacterial infections has begun to emerge [16–19]. Recently, several manuscripts identified that type I IFNs can exert effects in the regulation of immune and tissue homeostasis upon bacterial insult that may have beneficial or detrimental consequences for the host [20–23]. *S*. *aureus*, as well as other pathogen-associated molecular patterns (PAMPs), enhance IFNα and IFNβ secretion *in vitro* and *in vivo* during infection via activation of intracellular PRRs [12,13,16,24]. *S*. *aureus* infection has been shown to induce varying levels of IFNβ production amongst the various strains of the bacteria [23,25]. Interestingly, Kaplan et. al. have shown that the USA300 strain of methicillin resistant *S*. *aureus* (MRSA) inhibits the expression of IFNβ in the skin and that treatment of mice with recombinant IFNβ decreases skin bacterial loads [12]. Secreted IFNα and IFNβ binds to the heterodimeric interferon-alpha/beta receptor (IFNAR) and results in JAK1/Tyk2-mediated phosphorylation and dimerization of STAT-1, STAT-2, and IRFs [22].

STAT-1 activation is required for optimal skin host defense against *S*. *aureus* [9,26]. However, STAT-1 enhances the expression of the JAK/STAT inhibitor suppressor of cytokine signaling-1 (SOCS-1) [11,27] that could potentially inhibit inflammation and host defense. We and others have shown that SOCS-1 inhibits STAT-1 dependent MyD88 expression in macrophages [28,29]. We have also shown that SOCS-1 inhibits glycolysis and inhibits inflammatory responses during sepsis [28]. Whether SOCS-1 influences skin host defense remains to be elucidated. SOCS-1 acts mainly through direct inhibition of the JAK tyrosine kinase to prevent STAT-1 phosphorylation and activation in a classical negative feedback loop [30]. The SOCS-1 protein has three functional domains: a kinase inhibitory region (KIR), an SH2 domain, and a SOCS box. The KIR domain functions as a pseudo-substrate that inhibits JAK2-mediated STAT-1 and STAT-3 phosphorylation and activation. The SH2 domain binds directly to the activation loop of JAK2, to allow for this blocking of STAT activation [30,31]. The SOCS box targets proteins such as JAK2, MyD88 adaptor like protein (MAL) for degradation by the ubiquitin-proteasome pathway through recruitment of the E3 ubiquitin ligase scaffold Cullin 5 and other components of the E3 ubiquitin ligase complex [32,33].

In addition to STAT-1, SOCS-1 also prevents the activation of different transcription factors, such as NF-κB and AP-1 [29,34,35]. SOCS-1 can also inhibit phagocyte function via 1) hampering the TLR-MyD88-dependent activation of NF-κB by targeting MyD88-adaptor-like protein (MAL) [36]; 2) inhibiting IL-1 receptor-associated kinase (IRAK) [33]; 3) preventing MAPK signaling by binding to apoptosis signal-regulating kinase 1 (ASK-1) [34]. Since SOCS-1 exerts pleiotropic effects in phagocytes, it is expected that SOCS-1 could influence host defense. Indeed, SOCS-1 has demonstrated a detrimental impact in different infections, including viral, fungal, parasitic, and bacterial infections [20–22]. However, whether SOCS-1 is an important component of *S*. *aureus* skin host defense remains to be determined.

We hypothesize that SOCS-1 negatively impacts *S*. *aureus* skin infection outcomes by limiting phagocyte host defense and the inflammatory response. Using a combination of *in vivo* bioluminescent imaging along with pharmacological (SOCS-1 blocking peptide) and genetic (myeloid-specific SOCS-1 deletion) approaches, we identified a heretofore unknown role for SOCS-1 as a negative regulator of skin host defense in both homeostatic and hyperglycemic conditions. Our data also sheds light on the potential role of SOCS-1 inhibition as a host-directed therapeutic opportunity to treat antibiotic-resistant pathogens by redirecting the inflammatory response and increasing antimicrobial effector functions of phagocytes.

## Methods

### Ethics statement

Mice were maintained according to National Institutes of Health guidelines for the use of experimental animals with the approval of Vanderbilt University Medical Center (protocol #M1600215) and Indiana University (#10500) Committees for the Use and Care of Animals. Experiments were performed following the United States Public Health Service Policy on Humane Care and Use of Laboratory Animals and the US Animal Welfare Act.

### Animals

C57BL/6J breeding pairs were initially obtained from the Jackson Laboratory and maintained by breeding at Vanderbilt University Medical Center (VUMC), Nashville, TN, USA. MMDTR or *Csfr1*^*LsL-DTR-mCherry*^*_LysM*^*Cre*^ mice were generated by breeding the *Csf1r*-HBEGF/mCherry) 1Mnz/J plus *LysM*^*cre/cre*^ mice as previously reported [37] and allowed for the detection of monocytes/macrophages by mCherry fluorescence. EGFP-LysM mice were donated by Dr. Nadia Carlesso (City of Hope, Duarte, CA, USA). Catchup^IVM-red^ mice [38] were donated by Dr. Matthias Gunzer (Institute for Experimental Immunology and Imaging, University Hospital, University Duisburg-Essen, Essen, Germany). Wild-type BALB/c and IFNAR -/- mice were a gift from Dr. Stokes Peebles (Vanderbilt University Medical Center- VUMC). C57BL/6 *Socs1*^*fl*^ mice were obtained from Warren Alexander (Walter and Eliza Hall Institute, Parkville, Victoria, Australia) [39], and this strain was crossed with LysM^cre/cre^ mice (Jackson Laboratory) to create SOCS1^Δmyel^ mice. C57BL/6 *Socs1*^*fl*^ littermates were used as controls.

### Induction of hyperglycemia

For streptozotocin (STZ)-induced hyperglycemia, 6- to 8-week-old male C57BL/6J mice were treated by i.p. injection with 40 mg/kg of STZ (Adipogen) dissolved in 0.1 M sodium citrate buffer once daily for 5 consecutive days [40]. Euglycemic control mice received on the vehicle control, citrate buffer. Mice were considered hyperglycemic when blood glucose levels were >250 mg/dl. Mice were treated with STZ to induce hyperglycemia 30 days before MRSA skin infection.

### *S*. *aureus* strains

The MRSA USA300 LAC strain was a gift from Bethany Moore (University of Michigan, Ann Arbor, Michigan, USA [41]. The bioluminescent USA300 (NRS384 lux) strain was a gift from Roger Plaut (Food and Drug Administration, Silver Spring, Maryland, USA [42]). The methicillin-susceptible *S*. *aureus* (MSSA) Newman strain was obtained from Eric Skaar (Vanderbilt University Medical Center, Nashville, TN). The GFP-expressing USA300 LAC strain was a gift from William Nauseef (University of Iowa, Iowa City, Iowa) [43]. MRSA stocks were stored at –80°C. MRSA was cultured as previously described [44].

### MRSA skin infection and treatments

The murine skin infection model was adapted from a previous study [44]. Male mice between 6 and 12 weeks of age were used for MRSA skin infection. Mice were infected with approximately 3 × 10^6^ MRSA, and biopsies and sample collection were taken at various times, ranging from 6 hours to 9 days after infection, as previously described [26,44]. Lesion size was measured daily via caliper, and the affected area was calculated using the standard equation for the area (length x width) [45]. The iKIR (DTHFRTFRSHSDYRR) and scrambled-KIR peptide control (DTHFARTFARSHSDYRRI) were obtained from GenScript [28]. A lipophilic palmitoyl group was added to the N-terminus of both sequences to facilitate cell penetration [31]. Mice were treated intraperitoneally with 50 μg of either the iKIR peptide or the scrambled peptide control [28] 1 hour prior to infection and once-daily following infection. Mice were injected intraperitoneally with 40mg/kg of the anti-IFNGR or IFNAR antibody 3 hours before infection, followed by infection for 24 hours.

### Skin biopsy collection and determination of *in vivo* bacterial load

8 mm punch biopsies were collected from naive and infected skin at different time points post-infection and used to determine bacterial counts, histological analysis, cytokine production, mRNA expression, and flow cytometry analysis [26]. For bacterial counts, skin biopsy samples were collected, weighed, processed, and homogenized in tryptic soy broth (TSB) media. Serial dilutions were plated on tryptic soy agar. Colony-forming units (CFUs) were counted after incubation overnight at 37°C and corrected for tissue weight. Results are presented as CFU/g tissue.

### Histopathology analysis

For histological analysis, 8 μm skin sections were stained with Hematoxylin and eosin or gram staining to visualize bacteria in the infected skin, as we have previously shown [26]. Images of tissue sections were visualized and acquired using the Nikon Eclipse Ci and Nikon Ds-Qi2 (Nikon, Tokyo, Japan).

### *In vivo* bioluminescence imaging (BLI) and analysis with IVIS

An IVIS Spectrum/CT (Perkin Elmer) *in vivo* optical instrument was used to image bacterial bioluminescence and phagocyte fluorescence in the mice. Bioluminescence and fluorescent imaging and analysis were performed as previously described [46].

### Skin single cell isolation and staining for flow cytometry

Skin biopsy specimens were collected and minced before digestion in 1mL of DMEM with 1 mg/ mL collagenase D (Roche Diagnostics) for 3 hours at 37°C. Single-cell suspensions were treated with CD16/32 Fc blocking antibodies (Biolegend; catalog 101310; clone 93) to prevent non-specific antibody binding and stained with the fluorescent-labeled antibodies for 20 minutes followed by fixation using 1% paraformaldehyde. The following antibodies were utilized: F4/80-FITC (Biolegend; catalog 123107; clone BM8), Ly6G-PerCP/Cy5.5 (Biolegend; catalog 127616; clone 1A8) Ly6C-AF647 (Biolegend; catalog 128010; clone HK14), CD11b-PE/Cy7 (Biolegend; catalog 101216; clone M1/70). Analyses were completed using FlowJo software (FlowJo, Ashland, OR).

### Detection of cytokines and chemokines

Biopsy samples were collected, weighed, and homogenized in TNE cell lysis buffer containing phosphatase and protease inhibitors and centrifuged to remove cellular debris. Skin biopsy homogenates were then analyzed using the pro-inflammatory-focused 18-plex Discovery Assay from Eve Technologies (Eve Technologies, Calgary, AB) to detect cytokines and chemokines. Levels of IFNγ, IFNα, and IFNβ were measured the time points indicated in the legends by ELISA, (IFNγ -Invitrogen #88–7314) (IFNα-Biolegend #439407) (IFNβ-PBL #42120–1). Protein concentration was corrected for tissue weight.

### Detection of reactive oxygen and nitrogen species

*In vivo* and *in vitro* nitrate/nitrite levels were measured using a modified Griess assay (Sigma Aldrich #G4410) following manufacturers’ protocol. *In vivo* hydrogen peroxide (H_2_O_2_) production was measured with the Amplex Red Assay (ThermoFisher #A2218) as we have previously shown [47].

### Immunoblotting

Western blots were performed as previously described [29]. Protein samples were resolved by SDS-PAGE, transferred to a nitrocellulose membrane, and probed with commercially available primary antibodies against SOCS-1, total-STAT-1, phosphorylated STAT-1 (Y701), total- and phosphorylated STAT-3 (S727) (all at 1:1000; Cell signaling), or β-actin (1:10,000; Invitrogen). Membranes were then washed and incubated with appropriate fluorophore-conjugated secondary antibodies (1:10,000, anti-rabbit IgG, IRDye 800CW antibody, #926–32211, Licor). Relative band intensities were quantified using ImageJ software (NIH), as previously described [29].

### RNA isolation and quantitative real-time PCR

Skin biopsies samples were collected, and total RNA was isolated using lysis buffer (Buffer RLT; QIAGEN) following the manufacturer’s protocol. The RT^2^ First Strand Kit reverse transcription system (QIAGEN) was used for cDNA synthesis, and quantitative PCR (qPCR) was performed on a CFX96 Real-Time PCR Detection System (Bio-Rad Laboratories). Relative gene expression was calculated using the comparative threshold cycle (C_t_) and expressed relative to control or WT groups beta actin (ΔΔCt method). Primers for β-actin *and Socs1* were purchased from Integrated DNA Technologies (IDT, Coralville, IA).

### Nanostring and gene enrichment analysis

Global gene expression in infected skin biopsies from wild-type hyperglycemic and euglycemic mice was assessed by the NanoString nCounter gene expression system (Nanostring, Seattle, WA). Mouse Myeloid Innate Immunity panel for 770 genes in 19 different pathways was used for the analysis. Designed CodeSet underwent extensive quality control to avoid cross-hybridization to non-target molecules in samples. RNA was extracted from the infected skin using Trizol. The purity and concentration of the RNA were confirmed spectrophotometrically with a Nanodrop (Thermo Scientific, Waltham, MA). 100 ng of RNA (20ng/μl) was hybridized with probe CodeSet before running samples on NanoString. nSolver 3.0 software used to assess the quality of the run, followed by the deletion of the low-quality sample from further analysis. Two-sided hypergeometric statistical analysis was performed with the Kappa Score threshold setting of 0.3. Enrichment depletion was calculated based on Benjamini-Hochberg Correlated p-values of <0.05.

### Bone marrow-derived macrophage generation

Bone marrow cells were flushed from both tibias and femurs of mice with ice-cold PBS. Cells were centrifuged, and red blood cells were lysed using ACK buffer. Cells were adjusted to 1x10^6^ cells/mL in DMEM with FBS (5%), M-CSF (20ng/mL), and GM-CSF (10ng/mL) and maintained at 37°C with 5% CO_2,_ and the cell culture media containing M-CSF and GM-CSF was changed every 3 days until day 7 when the cell was fully differentiated.

### Phagocytosis and killing assays

Bacterial phagocytosis and killing were performed as previously shown [26,28,29,47]. Briefly, BMDMs (2 × 10^5^/well) were plated into two individual 96-well plates with opaque walls and clear bottoms. Cells were pretreated with 10 μM iKIR or scrambled (SCR) KIR peptide for 1 hour before the addition of GFP-MRSA at a multiplicity of infection of 50:1 [26]. Infected cells were incubated 1 hour to allow phagocytosis, and both plates were washed with warm PBS, and GFP fluorescence was measured on the first plate. The second plate was then maintained in PBS with SCR KIR or PBS with iKIR peptide and was incubated for another 2 hours for killing assays. To determine the role of NO in iKIR-mediated microbial killing, cells were treated with 50 μM of the iNOS inhibitor 1400W dihydrochloride (Tocris). To measure the intensity of intracellular GFP fluorescence, extracellular fluorescence was quenched with 500 μg/mL trypan blue, and the GFP fluorescence was quantified using a fluorimeter plate reader. Trypan blue served as a blank. A reduction in GFP fluorescence in the killing plate relative to the phagocytosis plate indicated bacterial killing [26,48].

### Statistical analysis

Results are shown as a mean +/- SEM and were analyzed using GraphPad Prism 8.0 software (GraphPad Software, San Diego, CA). For comparisons between two experimental groups, a Mann-Whitney test was used, and for comparisons among three or more experimental groups, one-way ANOVA followed by Bonferroni multiple comparison test was used. P < 0.05 was considered significant.

## Results

### SOCS-1 impairs skin host defense

**Fig 1:**
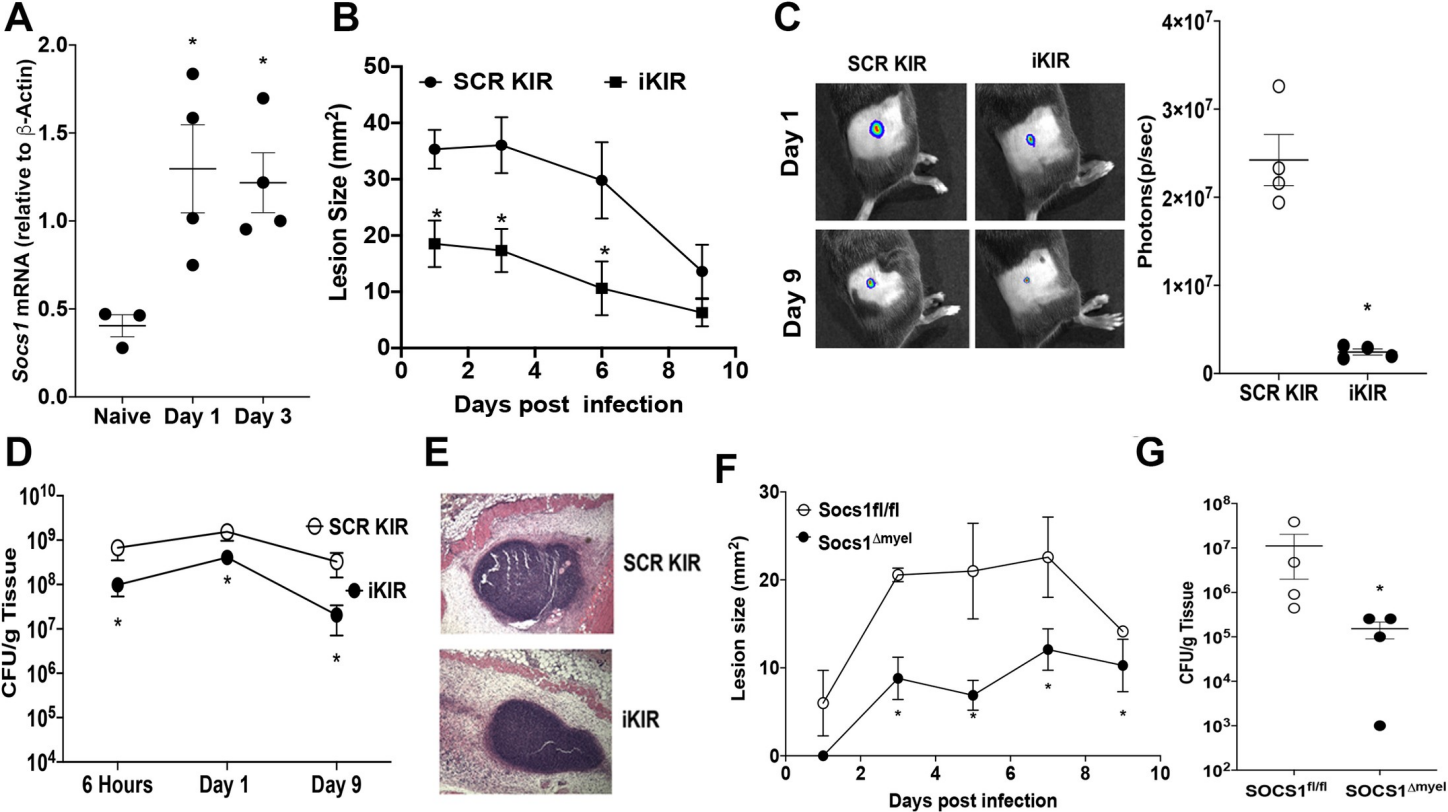
Inhibition of SOCS-1 actions improves subcutaneous skin infection outcome. **A)** mRNA expression of *Socs1* in the skin of mice infected subcutaneously with MRSA at day 1 and day 3 post-infection as determined by qPCR. **B)** Bioluminescent infection area in mice treated with SCR or SOCS-1 iKIR peptide using the *in vivo* animal imaging (IVIS Spectrum) detection system. **C) Right–**Total flux (photons/sec) of bioluminescent MRSA detected in mice treated as in **B** using IVIS Spectrum. **Left–**Representative images of bioluminescent MRSA in the skin of mice treated as in **B** using planar bioluminescent imaging. **D)** Bacterial load determined by CFU measured in the skin biopsy homogenates from mice treated as in **B** determined at the indicated time points after infection. **E)** Representative H&E stains from mice treated as in **B** and shown at 10X magnification **F)** Infection area measured every other day for 9 days post-infection in SOCS1^fl^ and SOCS1^Δmyel^ mice. **G)** Bacterial load determined by CFU measured in the skin biopsy homogenates in SOCS1^fl^ and SOCS1^Δmyel^ mice at day 9 post-infection. Data represent the mean ± SEM from 3–9 mice from 2–3 independent experiments. *p < 0.05 vs. SCR KIR.

To explore the role of SOCS-1 in *S*. *aureus* skin infection, we initially examined *Socs1* mRNA expression in the MRSA-infected skin of C57BL6/J mice. We observed a gradual increase in skin *Socs1* mRNA expression and protein levels at days 1 and 3 post-infection (Figs 1A and S1A). Next, we investigated the consequences of SOCS-1 inhibition in skin host defense using pharmacologic and genetic approaches. SOCS-1 binds to JAK receptors via its SH2 domain and a short motif upstream of SH2, known as the KIR (kinase inhibitory region) that directly inhibits the kinase activity of JAK tyrosine kinases. The KIR of SOCS-1 occupies the substrate binding groove of JAK and prevents subsequent phosphorylation. Previously, we have utilized a SOCS-1 KIR blocking peptide termed iKIR that enhanced STAT-1 and STAT-3 phosphorylation in macrophages to promote cytokine storm during sepsis [28]. Here, we examined if this peptide could influence skin host defense. During infection with bioluminescent MRSA, wild-type mice were treated 1 hour before infection with the iKIR peptide or scrambled (SCR) KIR peptide control [28,31,49] followed by daily treatments post-infection. Mice treated with iKIR showed decreased infection area and bacterial loads when compared to infected mice treated with the scrambled control KIR (Fig 1B and 1C), as evidenced by decreased bacterial bioluminescence, as well as decreased CFUs (Fig 1D). The beneficial effect of iKIR treatment is not restricted to MRSA as we also observed that iKIR treatment decreased bacterial load during infection with the MSSA Newman strain (S1B Fig). Interestingly, iKIR treatment decreased bacterial burden in the skin of mice as early as six hours post-infection that persisted over the course of a nine-day infection (Fig 1D) but did not appear to directly impact bacterial viability or growth during axenic cultures (S1C Fig). Lower bioluminescent signals also correlated with a more compact abscess in iKIR-treated mice (Fig 1E).

SOCS-1 regulates both the adaptive and innate immune response [30]. As neutrophils and macrophages are required to control MRSA skin infection [5,6], we further studied the role of myeloid-specific SOCS-1 actions during skin infection. Infection in *Socs1*^Δmyel^ mice resulted in smaller lesion size over time and decreased bacterial burden in the skin at day 9 post-infection (Fig 1F and 1G). Together, these data suggest that phagocyte SOCS-1 expression negatively impacts MRSA skin infection outcomes.

### SOCS-1 inhibition increases bacterial phagocytosis and killing in macrophages

**Fig 2:**
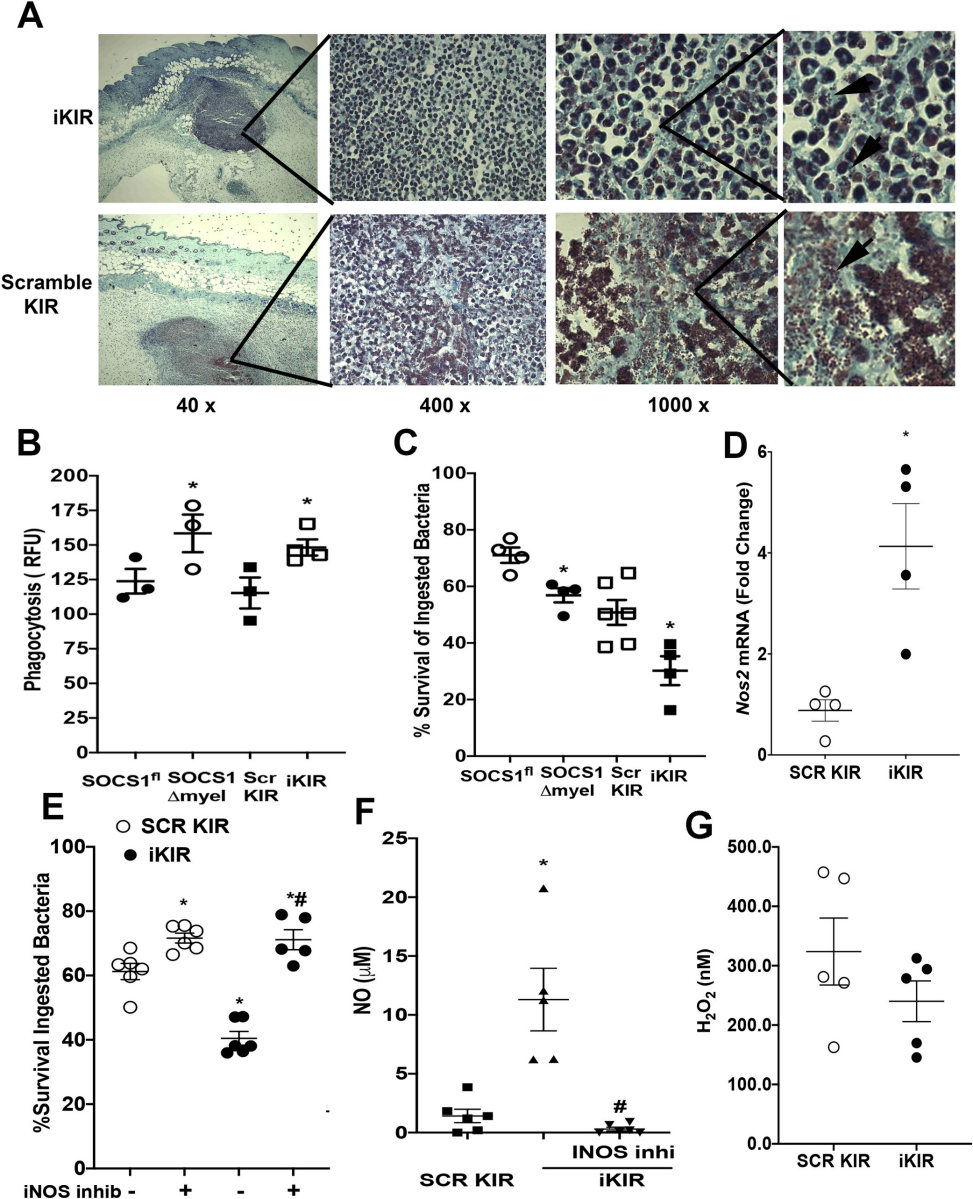
SOCS-1 inhibition enhances antimicrobial effector functions in BMDMs. **A)** Gram staining of skin biopsies collected at day 1 post-MRSA skin infection from iKIR and scrambled KIR treated mice. Gram staining to label gram-positive bacteria is shown in purple/brown. Magnifications are as shown. Black arrows indicate extracellular MRSA clusters. Images are representative of 3–5 mice per group. **B)** Phagocytosis of GFP tagged MRSA by BMDM’s from SOCS1^fl^ and SOCS1^Δmyel^ mice or BMDM’s from WT mice treated with the SCR KIR or iKIR peptide. **C)** Determination of Bacterial killing of GFP tagged MRSA by BMDMs from **B** as described in Phagocytosis and Killing assays—Methods. **D)** mRNA expression of *Nos2* in the skin of infected mice treated with either SCR KIR or iKIR peptide at day 1 post-infection as determined via qPCR. **E)** Determination of bacterial killing of GFP-tagged MRSA as in **C** with BMDMs from WT mice treated with either SCR KIR, iKIR, or iKIR+ an iNOS inhibitor (1400W dihydrochloride). **F)** Measurement of nitric oxide in the supernatant of BMDMs from **E**. **G)** H_2_O_2_ levels in the skin of mice treated with either SCR KIR or iKIR at day 1 post infection as determined by Amplex Red assay. Data represent the mean ± SEM from 3–6 mice from 2–3 independent experiments. *p < 0.05 vs. SOCS1^fl^ or SCR KIR treated mice. #p < .05 vs. iKIR treated mice.

Due to the impact of SOCS-1 inhibition on bacterial clearance in the skin, we hypothesized that SOCS-1 might inhibit macrophage antimicrobial effector functions, such as phagocytosis and bacterial killing. We next examined Gram stains in the infected skin sections of wild-type mice treated with iKIR or scrambled KIR to determine whether differences in bacterial ingestion, niche location, and burden were evident between iKIR-treated or scrambled KIR-treated animals. MRSA was found mostly within cells in iKIR-treated mice, while a higher abundance of extracellular bacteria was observed in the skin of scrambled KIR-treated animals at day 1 post-infection (Fig 2A). When bone marrow derived macrophages (BMDMs) from both *Socs1*^Δmyel^ mice and wild-type mice treated with the iKIR peptide were challenged with GFP-MRSA, we observed enhanced bacterial phagocytosis when compared to *Socs1*^*fl*^ BMDMs, or SCR KIR treated cells (Fig 2B). Importantly, SOCS-1 inhibition enhanced bacterial killing in BMDMs (Fig 2C). These data suggest that SOCS-1 is a negative regulator of macrophage antimicrobial effector functions that might be involved in MRSA skin infection control.

*S*. *aureus* is sensitive to killing via nitric oxide (NO) [5,9,50]. To identify if SOCS-1 targets NO production in macrophages, we initially determined *Nos2* expression (the gene that encodes the inducible nitric oxide synthase protein) (iNOS) in iKIR treated and infected mice. We found significantly higher *Nos2* expression in the skin of iKIR-treated animals than mice treated with the scrambled peptide control at day 1 post-infection (Fig 2D). As increased NO release could lead to more efficient bacterial killing *in vivo*, we determined whether increased iNOS/NO in iKIR treated mice accounts for improved microbial killing. BMDMs were treated with iKIR and an iNOS inhibitor (1400W dihydrochloride), followed by bacterial killing determination. iNOS inhibition ablated the beneficial effects of SOCS-1 inhibition on bacterial killing (Fig 2E). Notably, SOCS-1 inhibition increased NO release in MRSA-challenged BMDMs, and the treatment with the iNOS inhibitor reversed this trend (Fig 2F). Furthermore, when we examined whether SOCS-1 could regulate the production of the reactive oxygen species (ROS) known to be involved in *S*. *aureus* killing, we did not detect differences in skin H_2_O_2_ production between scrambled KIR and iKIR-treated and infected mice (Fig 2G). Together these data demonstrate a role for SOCS-1 in regulating antimicrobial programs in macrophages dependent on NO release.

### SOCS-1 inhibits STAT-1 dependent production of pro-inflammatory cytokines and neutrophil recruitment during skin infection

**Fig 3:**
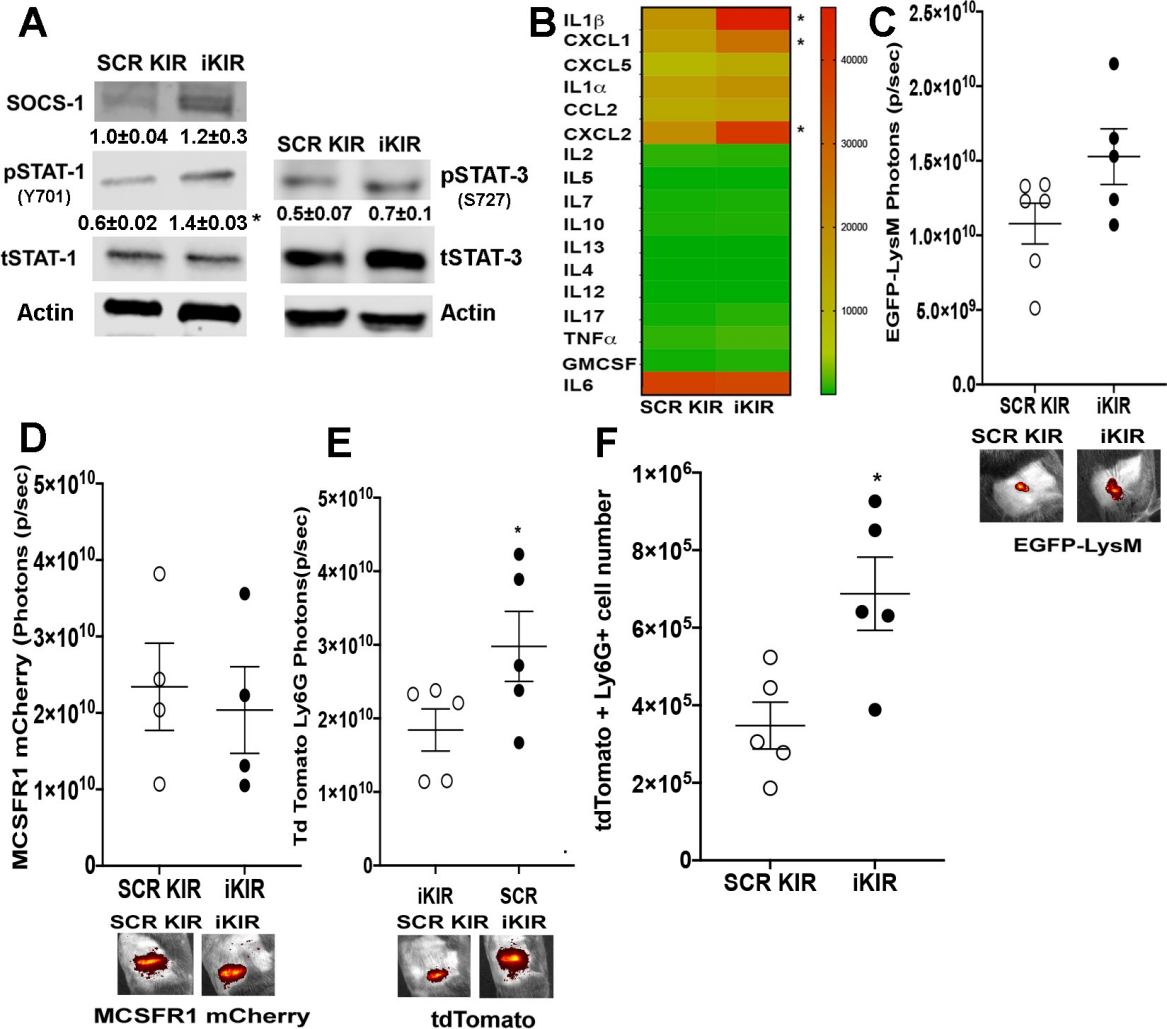
SOCS-1 inhibition increases pro-inflammatory cytokines and immune cell recruitment during skin infection. **A)** Representative Western blots for SOCS-1, pSTAT-1 (Y701), tSTAT-1, pSTAT-3 (S727), tSTAT-3, and Actin from biopsies collected at day 3 post-infection in SCR KIR and iKIR treated animals. The numbers represent mean densitometry analysis of the bands ± SEM (n = four to five mice/group). **B)** Heat-map of proteins involved in the inflammatory immune response and its resolution in mice treated with either iKIR or SCR KIR at day 1 post-infection as measured using bead array multiplex (Eve Technologies). Proteins are listed on the left-hand *y*-axis, grouped alphabetically in clades. Red indicates higher abundance, whereas green represents lower abundance. Each column for each condition represents a technical replicate (*n* = 4-5/group). **C)** Total flux (photons/sec) of EGFP detected in EGFP-LysM mice at day 3 post infection treated with either SCR or iKIR. Representative pictures are shown below. **D)** Total flux of mCherry signal detected in MMDTR mice at day 3 post infection treated with either SCR or iKIR, representative pictures are shown below. **E)** Total flux of tdTomato signal detected in Catchup^IVM-red^ mice at day 3 post infection treated with either SCR or iKIR with representative picture shown below. **F)** Total number of tdTomato+ cells in biopsies collected from mice in **E.** Data represent the mean ± SEM from 3–5 mice from 2–3 independent experiments. *p < 0.05 vs. SCR KIR treated mice.

Next, we examined the impact of SOCS-1 inhibition on the inflammatory milieu during skin infection. Initially, we determined whether SOCS-1 inhibition influenced STAT-1 activation *in vivo*. We found that while *S*. *aureus* infection induces STAT-1 phosphorylation (Y701), iKIR treatment further enhanced STAT-1 phosphorylation in the infected skin compared to mice treated with the scrambled KIR control (Fig 3A). Importantly, iKIR did not change SOCS-1 expression or impact STAT-3 phosphorylation (S727) during MRSA infection (Fig 3A). We also confirmed that skin infection in *Socs1*^Δmyel^ mice increased pSTAT-1 abundance compared to infected wildtype control animals. These data suggest that the SOCS-1/STAT-1 signaling axis is a component of skin host defense.

We then determined whether iKIR enhances the production of 17 cytokines and chemokines known to modulate the skin inflammatory response and host defense [5]. Our data show that iKIR enhances specifically the production of pro-inflammatory cytokines known to drive proper abscess formation and improved infection outcome (IL-1β) [3], neutrophil recruitment (CXCL1 and CXCL2) [51], but not monocyte recruitment (CCL2) [52] in response to MRSA skin infection (Figs 3B and S2A). These data suggest that SOCS-1 controls a specific group of cytokines and chemokines known to influence neutrophil migration and functions to prevent an efficient host immune response to MRSA skin infection.

Since IL-1β, CXCL1, and CXCL2 drive phagocyte recruitment during skin infection [52] and iKIR enhances the abundance of these mediators at days 1 and 3 after infection (Figs 3B and S2B), we aimed to investigate whether iKIR treatment increases neutrophil and/or monocyte-derived macrophage migration to the site of infection. To examine specific phagocyte recruitment, we detected the accumulation of specific phagocytes using different transgenic mice that express fluorescent proteins specifically in myeloid cells, monocytes/macrophages, and neutrophils using IVIS optical imaging [37,38,53]. We first infected and treated EGFP-LysM mice (mainly neutrophils as well as monocytes and macrophages express EGFP) [53]. We detected a non-significant increase in GFP signal in the skin of iKIR-treated mice when compared to infected animals treated with the scrambled control (Fig 3C). To examine whether iKIR influences monocyte and macrophage recruitment, we infected and treated MMDTR mice (CSFR1+ cells express the mCherry fluorescent protein) and also found no differences between SCR KIR and iKIR treated animals (Fig 3D). To determine if iKIR specifically influences neutrophil recruitment, we infected and treated Catchup^IVM-red^ mice in which Ly6G^+^ cells express the tdTomato fluorophore. We found iKIR treatment greatly increased the tdTomato signal during infection (Fig 3E). We confirmed these findings by flow cytometry analysis where we did not detect differences in total macrophage (CD11b+ F4/80+) numbers but increased neutrophil abundance (CD11b+ tdTomato+) when compared to SCR KIR treated mice (Fig 3F). These data suggest that SOCS-1 is an endogenous inhibitor of neutrophil recruitment by hampering the secretion of chemoattractants such as CXCL1 an IL-1β that are involved in skin host defense [7,53].

### SOCS-1/type I interferon axis mediates skin host defense

**Fig 4:**
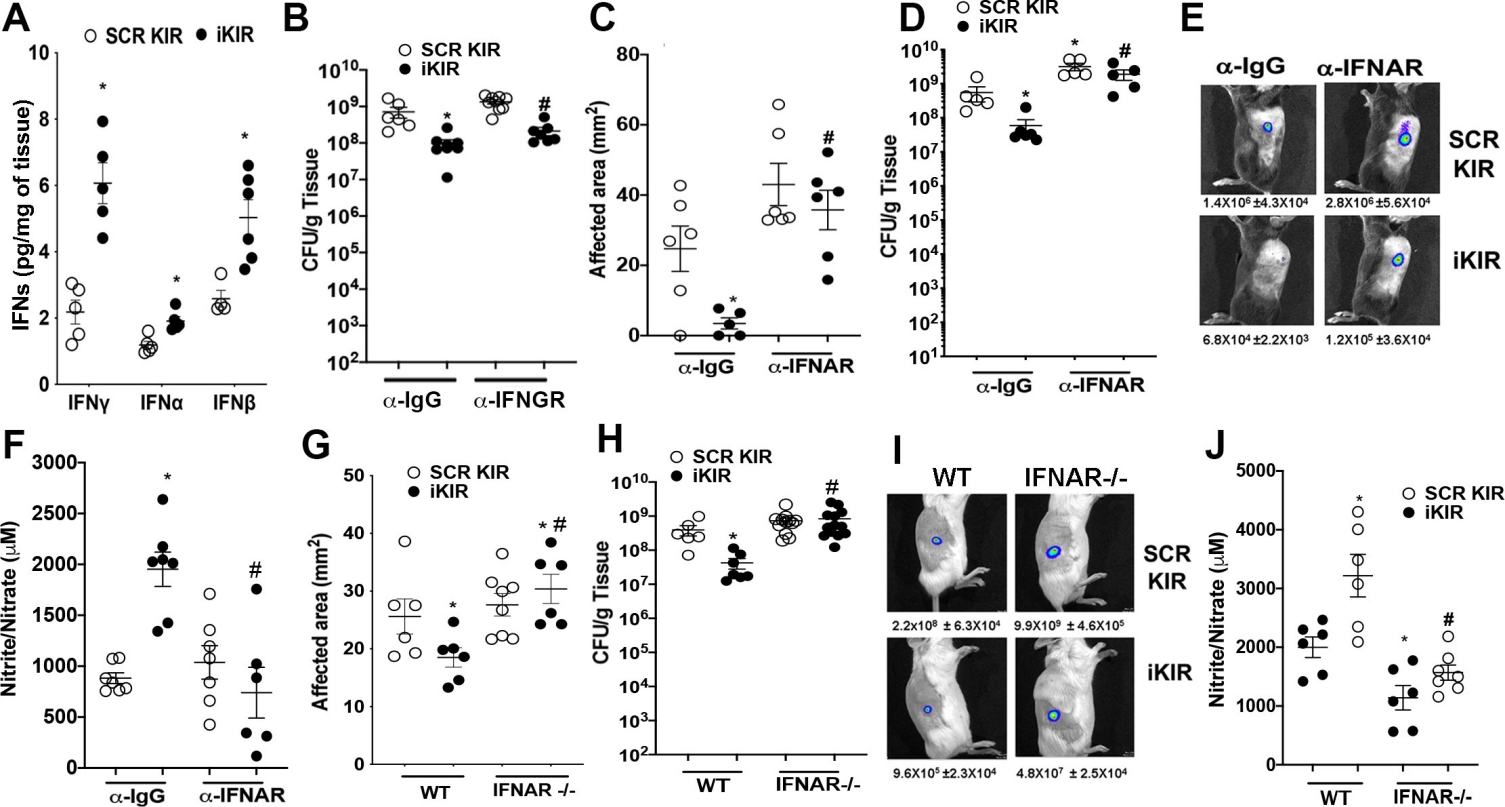
Inhibition of type I interferon signaling removes the benefit of SOCS-1 inhibition. **A)** Interferon levels in the skin at day 1 post-infection in SCR KIR and iKIR treated animals as measured via ELISA. **B)** Bacterial load as determined via CFU in skin biopsy homogenates of SCR KIR and iKIR treated mice treated with either IFNGR antibody or IgG control. **C)** Bioluminescent infection area in the skin of SCR KIR and iKIR treated animals treated with or without an IFNAR blocking antibody at day 1 post-infection. **D)** Bacterial load determined by CFU measured in the skin biopsy homogenates from mice treated as in **C** at day 1 post-infection. **E)** Representative images of bioluminescent MRSA in the skin of mice treated as in **C** using planar bioluminescent imaging with average flux (photons/sec) below **F)** Nitrite/Nitrate as measured via Griess assay from biopsies collected from mice treated as in **C** at day 1 post-infection. **G)** Surface lesion size as measured via caliper at day 3 post-infection in BALB/c or BALB/c IFNAR -/- mice treated with either iKIR or SCR KIR. **H)** Bacterial load determined by CFU measured in the skin biopsy homogenates from mice treated as in **G** at day 3 post-infection. **I)** Representative images of bioluminescent MRSA in the skin of mice treated as in **G** using planar bioluminescent imaging with average flux (photons/sec) below. **J)** Nitrite/Nitrate as measured via Griess assay from biopsies collected from mice treated as in **I** at day 3 post-infection. Data represent the mean ± SEM from 3–9 mice from 2–3 independent experiments. *p < 0.05 vs. SCR KIR+αIGG or SCR treated WT mice. #p<0.05 vs. iKIR+ αIGG or iKIR treated WT mice.

SOCS-1 is part of a negative feedback loop between type I and type II IFN production and STAT-1 activation [30]. Whether SOCS-1 regulates the production of type I and type II IFNs during skin infection remains to be determined. We detected higher IFNγ, IFNα, and IFNβ levels in skin biopsies from iKIR-treated and infected mice than scrambled KIR-treated mice at day 3 post-infection (Fig 4A). IFNβ was also significantly increased in the supernatant of iKIR BMDMs co-cultured with MRSA (S3A Fig). Due to the prominent role of IFNγ in enhancing phagocyte antimicrobial effector functions [14], we first blocked IFNγ actions in iKIR-treated and infected mice using an anti-interferon gamma receptor (IFNGR) antibody or IgG control antibody before infection and treatment with either iKIR or scrambled KIR peptide. Mice treated with the anti-IFNGR antibody plus scrambled KIR showed higher bacterial burden than mice treated with the control antibody as early as day 1 post-infection (Fig 4B). However, while iKIR plus IgG control decreased bacterial load, the treatment of mice with anti-IFNGR antibody did not prevent iKIR effects on bacterial clearance, demonstrating that IFNγ is not solely involved in the inhibitory effects of SOCS-1 during MRSA skin infection (Fig 4B).

Next, we determined whether iKIR actions are dependent on IFNα/β using both an IFNAR blocking antibody and IFNAR^-/-^ mice. While the pretreatment of mice with an anti-IFNAR antibody and scrambled KIR increased bacterial skin load, the blocking antibody also impaired iKIR-mediated decreases in lesion size (Fig 4C) and bacterial burden (Fig 4D and 4E). Treatment with the IFNAR antibody also decreased *in vitro* bacterial killing of iKIR treated BMDM (S3B Fig). We then confirmed that type I interferons were a potential mediator of improved skin host defense during SOCS-1 inhibition in a genetic model. Wild-type and IFNAR^-/-^ mice were treated with either iKIR or scrambled KIR peptide, followed by MRSA skin infection. Our data showed that IFNAR^-/-^ mice had increased lesion size at day 3 post-infection (Fig 4G) and bacterial load compared to WT mice (Fig 4H and 4I). Furthermore, IFNAR^-/-^ mice were refractory to iKIR treatment (Fig 4G, 4H and 4I). Next, we aimed to link SOCS-1/type I IFN and NO in *in vivo* MRSA skin infection. Our data showed that SOCS-1 inhibition enhances NO *in vivo* and blocking both SOCS-1 and IFNAR actions restored NO levels to the levels observed in scrambled KIR-treated mice (Fig 4F and 4J). Together, these data show a previously unknown operative axis of SOCS-1 and type I IFNs mediating NO-mediated microbial killing during bacterial skin infection.

### SOCS-1 drives impaired skin host defense in hyperglycemic mice

**Fig 5:**
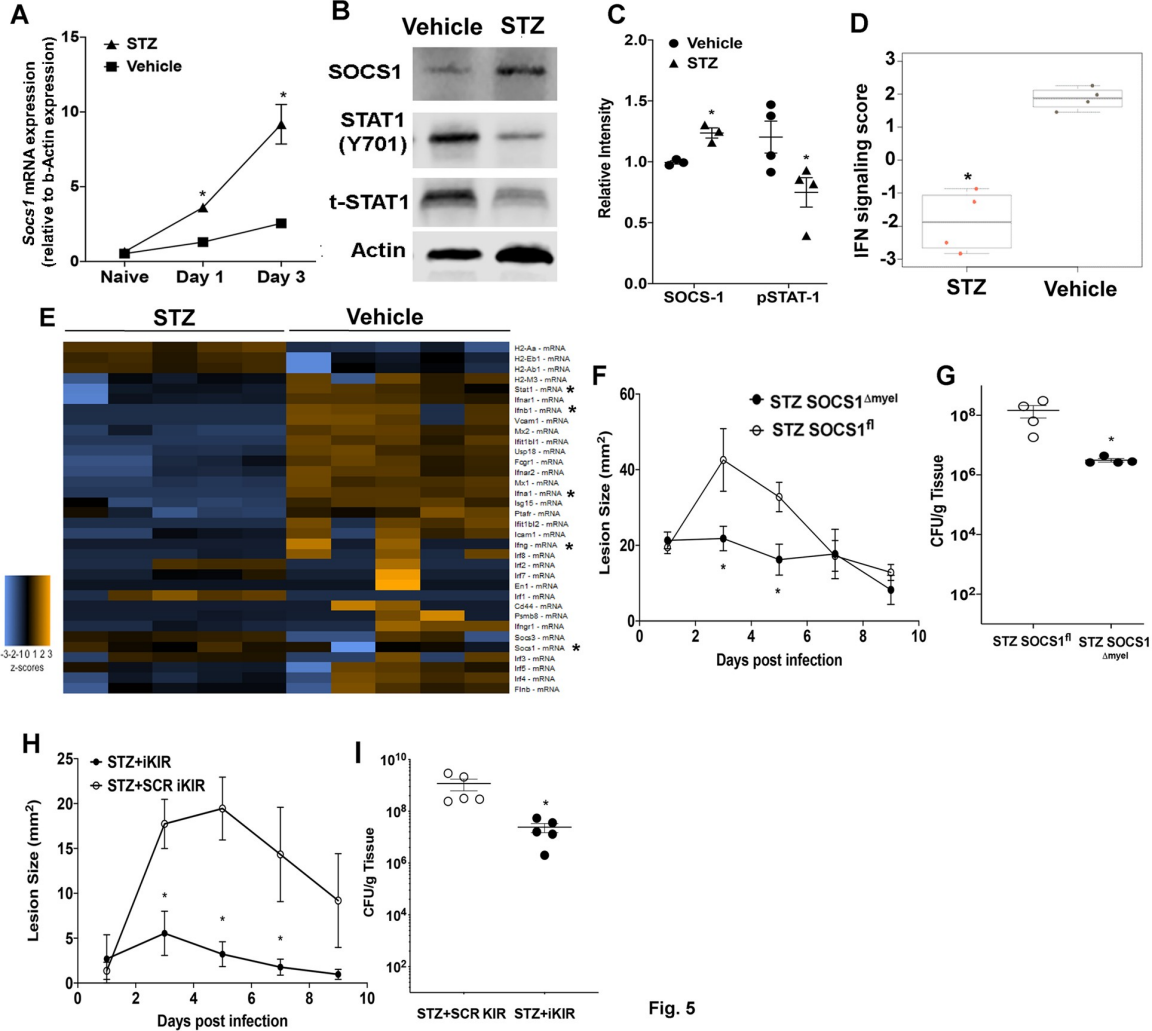
SOCS-1 impairs skin host defense in hyperglycemic mice. **A)** Expression of *Socs1* mRNA at day 1 and day 3 post-infection in biopsies collected from STZ induced hyperglycemic and control mice **B)** Representative Western blot for SOCS-1, pSTAT-1, and tSTAT-1 from biopsies collected at day 3 post-infection in STZ-induced hyperglycemic and control mice **C)** Densitometry quantification of separate Western Blots as shown in **B. D)** IFN signaling score determined using NanoSolver in mRNA isolated from infected skin of iKIR and scrambled KIR-treated mice. **E)** Heatmap of genes analyzed via NanoString from mRNA collected in **D**. **F)** Surface lesions size in STZ induced hyperglycemic and euglycemic SOCS1^fl^ and SOCS1^Δmyel^ mice over a 9 day subcutaneous MRSA infection. **G)** Bacterial load determined by CFU measured in the skin biopsy homogenates from mice treated as in **F** at day 9 post-infection. **H)** Surface lesions size in STZ induced hyperglycemic and euglycemic mice treated with either SCR KIR or iKIR over the course of a 9 day subcutaneous MRSA infection. **I)** Bacterial load determined by CFU measured in the skin biopsy homogenates from mice treated as in **H** at day 9 post-infection. Data represent the mean ± SEM from 3–6 mice from 2–3 independent experiments. *p < 0.05 vs. Vehicle controls, SOCS1^fl^ or SCR KIR treated mice.

Patients with hyperglycemia are more susceptible to MRSA skin infections than euglycemic individuals [44,54]. Therefore, we hypothesized that hyperglycemia enhances SOCS-1 expression and that blocking SOCS-1 actions might be a potential therapeutic approach to treat skin infections under hyperglycemic conditions. Initially, we determined *Socs1* mRNA expression over time in the skin of infected hyperglycemic and euglycemic mice. Our data show that *Socs1* expression was significantly upregulated in the skin of hyperglycemic animals at both day 1 and day 3 post-infection when compared to euglycemic animals (Fig 5A). Enhanced SOCS-1 expression correlated with decreased pSTAT-1 in skin biopsies of infected hyperglycemic mice (Fig 5B and 5C). Since SOCS-1 expression is increased in the skin of infected hyperglycemic mice, we asked whether the infection in hyperglycemic mice is characterized by an overall decrease in genes involved in IFN responses. Interestingly, our data show an overall downregulation of the IFN gene signature at day 1 post-infection in hyperglycemic mice (Fig 5D and 5E). When we examined specific IFN-related genes, we observed inhibition of *Ifna1*, *Ifnb1*, *Ifng*, and *Stat1* in the infected skin of hyperglycemic mice. Importantly, we observed increased *Socs1* (confirming the results in Fig 4A and 4B), *Socs3*, *Irf2*, *Irf3*, and *Irf7* (Fig 5E) in infected hyperglycemic mice. We then determined whether myeloid-specific SOCS-1 expression is involved in poor skin host defense in hyperglycemic mice. Our data show that hyperglycemic *Socs1*^*Δmyel*^ mice show decreased lesion size (Fig 5F) and bacterial load (Fig 5G) when compared to hyperglycemic *Socs1*^*fl*^ mice on day 9 post-infection. Next, we sought to investigate if iKIR could restore skin host defense and represent a potential therapeutic approach to treating antibiotic-resistant skin infections. Daily treatment of the hyperglycemic mice with iKIR significantly reduced lesions size and bacterial burden (Fig 5H and 5I). Together these data suggest that enhanced *Socs1* expression during hyperglycemia is detrimental to skin host defense and that iKIR treatment could represent host-directed immunotherapy to treat these antibiotic-resistant pathogens in the skin.

## Discussion

Monocytes, macrophages, and neutrophils are critical in the containment, elimination, and resolution of *S*. *aureus* infections in the skin and soft tissue. Due to the organ-specific diversity and pleiotropic function of these cells, by better understanding how these phagocytes respond to and eliminate pathogens, we might develop efficient therapeutic approaches to redirect the host’s immune response, improving microbial clearance while allowing for proper tissue repair. Host-directed immunotherapies are of particular importance, given the rapid rise of antibiotic resistance in several bacterial species, including *S*. *aureus* [55]. Furthermore, the rise of community-acquired MRSA (CA-MRSA) in healthy individuals outside of the healthcare setting [56], further confirms the dire need for host-directed immunotherapeutic strategies for these infections.

Many studies have demonstrated the potential of host-directed therapies to treat skin infections. Few examples include the treatment of mice with antimicrobial peptides [57], cytokines (IL-1β) [7], lipids (LTB_4_) [26], and enzyme inhibitors such as aspirin and indomethacin [58]. Here, employing pharmacological and genetic approaches, we demonstrate that SOCS-1 inhibition/deletion boosts different arms of the skin immune response and increases MRSA clearance. Furthermore, we also showed that the beneficial effects of SOCS-1 inhibition are not restricted to MRSA strains, as iKIR peptide treatment also increased bacterial clearance of the MSSA Newman strain, which indicates a common regulatory program controlled by SOCS-1 to enhance *in vivo* host defense.

SOCS-1 is a pleiotropic inhibitor of both the innate and adaptive immune response by controlling the actions of transcription factors and signaling effectors downstream of cytokine receptors, growth factors, and PRRs. We and others have shown that SOCS-1 inhibits MyD88 expression and actions [29,59,60]. SOCS-1 can prevent the differentiation of "pro-inflammatory" or M1 macrophages by limiting NF-κB p65 activation and STAT-1 actions [32]. Also, SOCS-1 inhibits TLR signaling by increasing MALD degradation and prevents MyD88 association with TLRs, thereby inhibiting the activation of downstream transcription factors [30]. Therefore, given the role of TLRs and inflammatory cytokines in *in vivo* host defense, it is anticipated that endogenous SOCS-1 might help prevent tissue damage and, consequently, impacts phagocyte antimicrobial effector function. Indeed, infections with both gram-positive [16,59], gram-negative [20] bacteria as well as *Mycobacterium tuberculosis* [18] and parasites, such as *Leishmania major* [61] and the fungus *Candida albicans* [62] promote SOCS-1 expression to actively suppress the immune response, allowing pathogen replication and immune evasion. Expression of SOCS-1 during infection correlates with reduced levels of the pro-inflammatory cytokines IL-1β, TNF-α, and IL-6 as well as antimicrobial NO and reactive oxygen species (ROS) [32,35]. However, the mechanisms of SOCS-1 actions in infected phagocytes remains to be fully determined. Here, we advanced the field forward by demonstrating that myeloid-specific SOCS-1 inhibits phagocyte antimicrobial effector function, neutrophil migration to the site of the infection, and ultimately linked SOCS-1 actions to IFNα/β *in vivo*.

SOCS-1 is known as a STAT inhibitor, and therefore, most of the reported SOCS-1 actions indicate a role in regulating gene transcription and therefore take hours/days to be detected. Here, we unveiled a new role for SOCS-1 in the early macrophage response, namely phagocytosis and microbial killing. It has been shown that SOCS-1 deletion amplifies *C*. *albicans* phagocytosis by controlling IFNγ actions [62]. In our current study, we separated the transcriptional outcomes of SOCS-1 vs. early signaling effects using the iKIR peptide. Although we did not further investigate the intracellular targets of SOCS-1 in *S*. *aureus* phagocytosis, we identified NO, but not ROS, as an intermediate of SOCS-1 actions in microbial killing. A specific role for SOCS-1 in inhibiting phagocyte antimicrobial effector function was also evidenced by the detection of intracellular bacteria in the iKIR-treated mice, while in scrambled KIR treated mice, most of the bacteria were observed extracellularly. Our future studies will address this critical question, and we expect to unveil the targets and mechanisms involved in SOCS-1 inhibition of phagocytosis.

Chemokines and IFNs enhance both the recruitment and antimicrobial effector functions of phagocytes during infection [52,63]. Recent work has also demonstrated a novel role for IFNγ in the activation of the fibrinolytic system to control abscess thickness, improve leukocyte recruitment, and drive abscess breakdown during *S*. *aureus* and *C*. *albicans* infection [8]. While IFNγ is a potent macrophage activator known to increase gene expression of many antimicrobial effectors such as members of the NADPH oxidase complex (gp91phox, p47phox), iNOS, and antimicrobial peptides, IFNγ also enhances the expression of MHCII and antigen presentation [14]. In our study, IFNγ production alone does not seem to account for the therapeutic benefit of SOCS-1 inhibition in skin host defense. Interestingly, SOCS-1 inhibition mimicked several aspects of the protective effects of IFNγ such as a highly organized and compact abscess with a thick capsule. If SOCS-1 regulates the expression of collagen and fibrinolytic proteins involved in abscess capsule formation remains to be determined.

Though the bulk of studies on IFNα and IFNβ focus on their role in anti-viral adaptive immunity, emerging studies demonstrate a role for IFNβ in macrophage activation and bacterial killing [22]. IFNβ signaling in inflammatory macrophages has been shown to act in an autocrine signaling manner to promote iNOS expression and NO release along with other pro-inflammatory mediators to drive increased bacterial killing [50,63,64]. These studies highlight that while IFNβ treatment alone might not be enough to drive these phenotypes, IFNβ treatment combined with TLR1/2 and 4 stimulation significantly enhances NO levels. This fits into our current study since TLR2 is an important pathogen recognition receptor in binding the lipoteichoic acids that make up the cell wall of MRSA [63]. The autocrine nature of IFNβ signaling in macrophages would also explain why inhibition of SOCS-1 significantly improved infection outcomes at time points as early as 6 hours post-infection. Therefore, it seems likely that increased iKIR-dependent IFNβ production and/or actions during MRSA skin infection are promoting increased NO production that is driving increased bacterial killing in our current study.

That different *S*. *aureus* strains, with distinct severities of infection, exhibit different capabilities to induce IFN α/β production has been previously shown [24,25]. Both Martin et al. [13] and Parker & Prince [23] have demonstrated that the capacity of *S*. *aureus* to potently induce IFNβ production is detrimental to lung host defense, leading to increased bacterial burden, tissue damage, and mortality of intranasally infected mice. Furthermore, Parker et al. showed that the pathogenicity of *S*. *aureus* lung pathology correlates with its capacity to induce IFNβ production [23]. However, Kaplan et. al. has shown that *S*. *aureus* actually suppresses IFNβ production during skin infection compared to other gram-positive pathogens. Furthermore, they showed that the addition of topical IFNβ enhanced skin bacterial clearance and chemokine production [12]. In contrast, we found that MRSA skin infection increases IFNα/β production and confirmed that IFNα/β demonstrate protective effects in the skin (Fig 4). Differences between our manuscripts may be due to the higher inoculum used in their work (1x10^7^ vs. 3x10^6^ used here), and the strain of *S*. *aureus* (Newman vs. MRSA USA 300). Furthermore, the contradicting role of IFNα/β between lung and skin host defense may be due to a number of factors such as different cellular sources of type I IFNs, different cells and receptors involved in bacterial recognition, and the tolerogenic environment of the lung [5,65]. A common thread between our findings in the skin and those in the lung is the correlation between increased CXCL1 production with increased type I IFN signaling and increased neutrophil recruitment to the site of infection [13,23,24]. However, while this increased neutrophil recruitment and abscess formation can be detrimental during lung infection, they have been shown to be critical in skin host defense [3,5,66,67].

If SOCS-1-mediated type I IFN actions are detrimental for other strains of *S*. *aureus* in different organs remains to be determined. Also, we did not specify whether SOCS-1 modulates IFNAR signaling directly, and whether it acts on downstream events. We speculate that SOCS-1 could be inhibiting the activation of different IRFs, such as IRF7 and IRF3, but more studies are needed to address further the potential mechanisms of SOCS-1 mediated IFNα/β production and/or signaling during infections. Our study shows that increased iKIR-mediated IFNβ production and/or IFNAR signaling is enough to promote sufficient bacterial killing in the absence of IFNGR signaling *in vivo*. However, due to the known role of IFNγ in macrophage activation, we cannot exclude a potential role for IFNγ in iKIR-treated mice, as we only determined microbial clearance in a single time point after infection.

Modulation of SOCS-1 activation by mimics or antagonists has been a topic of intense research [31,33]. Since JAK/STAT pathways are involved in a number of inflammatory diseases, there is an abundance of research focusing on the modulation of KIR actions in models of diabetes, atherosclerosis, EAE, and dermatological diseases [33,62]. Interestingly, KIR antagonists can influence both STAT-1 and STAT-3 in a disease specific manner. We have previously shown that iKIR enhances both STAT-1 and STAT-3 in a murine model of sepsis and that iKIR-mediated STAT-3 activation amplifies HIF1α-mediated glycolysis [28]. Here, although we observed a specific effect of iKIR on STAT-1, but not STAT-3 phosphorylation (Fig 3A), the fact that we do not detect changes in IL-6 or IL-17 (which require STAT-3 production and activation to be produced [68]), suggests a specific role for the iKIR/STAT-1 axis in improved skin host defense. However, as mentioned above, iKIR has been shown to improve STAT-3 phosphorylation in other models of treatment [28,31] so we cannot discount the possibility of other off target effects with iKIR treatment. Whether STAT-1 is a downstream target for iKIR-mediated improved host defense remains to be determined.

To explore SOCS-1 inhibition as a potential therapeutic strategy in a highly susceptible population, we sought to investigate iKIR peptide improves host defense in hyperglycemic mice. We found that SOCS-1 expression is increased in macrophages from hyperglycemic animals. The iKIR peptide significantly decreased bacterial burden and lesion size compared to hyperglycemic mice treated with the scrambled peptide control. We also confirmed the beneficial role of SOCS-1 deletion in myeloid cells in skin host defense of hyperglycemic mice. Interestingly, we also unveiled an overall decrease in the expression of genes involved in IFN production and signaling. The basis for such drastic event is unknown, but it could include potential epigenetic modifications in the genes involved in IFN response. Nonetheless, these findings are intriguing and exciting, since we would expect that increased SOCS-1 expression in macrophages should decrease an overall exacerbated inflammatory response, but instead, we and others have shown that infection in hyperglycemic mice is characterized by a robust and detrimental migration of neutrophils and a delayed clearance of microbes [44,46]. In this context, aberrant intracellular SOCS-1 might be involved in preventing bacterial ingestion and killing while failing to regulate cytokine-dependent tissue inflammation. The mechanisms underlying SOCS-1 effects during infection in hyperglycemia/diabetes models are the current focus of our laboratory. Together this data suggests that SOCS-1 may be a potential target for future therapeutic intervention in skin infections and may benefit not only to highly susceptible patient populations but also to more broad patient populations with other bacterial infections of the skin.

## Supporting information

S1 Fig SOCS1 expression and effects in bacterial growth *in vivo* and *in vitro*. **A)** Representative immunoblotting of SOCS-1 and beta actin from skin biopsies of mice infected s.c. with MRSA for 1, 3, and 9 days. **B)** Bacterial load in skin biopsies homogenates of SCR KIR and iKIR treated mice infected with the MSSA Newman strains at day 3 post infection as determined by CFU quantification. **C)** MRSA growth curve as determined via OD600 of bacteria cultured in either 10 μM of iKIR or SCR KIR in the indicated time points. Data represent the mean ± SEM from 3–4 mice from 2–3 independent experiments. *p < 0.05 vs. SCR KIR treated mice.
(TIF)

S2 Fig SOCS-1 iKIR enhances the abundance of skin neutrophil chemoattractants overtime. **A)** Detection of cytokines and chemokines in mice treated with either iKIR or SCR KIR at day 1 post-infection as measured using bead array multiplex (Eve Technologies). These results are derived from Fig 3B. **B)** CXCL2 and **C)** CXC1 abundance in skin biopsies from mice infected and treated as in **A** and collected at days 3 and 9 post infection. Data represent the mean ± SEM from 3–7 mice. *p < 0.05 vs. SCR KIR treated mice.
(TIF)

S3 Fig SOCS-1 iKIR-mediated type 1 IFN enhances macrophage bacterial killing. **A)** Detection of IFNβ in the supernatant of BMDMs from WT mice treated with the SCR KIR or iKIR peptide and co-cultured with MRSA for 24 h by ELISA. **B)** Determination of bacterial killing of GFP-tagged MRSA by BMDMs from WT mice treated with either SCR KIR or iKIR peptide and either an IFNAR blocking antibody or IgG control antibody as described in Phagocytosis and Killing assay—Methods. Data represent the mean ± SEM from 2 independent experiments. *p < 0.05 vs. SCR KIR treated. #p<0.05 vs. iKIR+ αIgG.
(TIF)
