# Supplementary figures and images for: SOCS-1 inhibition of type I interferon restrains Staphylococcus aureus skin host defense
Source: PLoS Pathog. 2021 Mar 10;17(3):e1009387. doi: 10.1371/journal.ppat.1009387 (PMC7984627; doi:10.1371/journal.ppat.1009387)

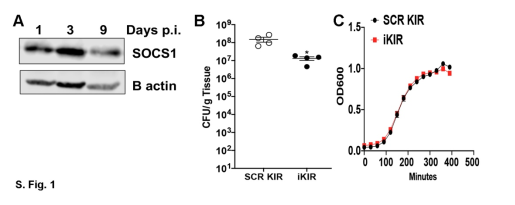

Supplement: S1 Fig — A) Representative immunoblotting of SOCS-1 and beta actin from skin biopsies of mice infected s.c. with MRSA for 1, 3, and 9 days. B) Bacterial load in skin biopsies homogenates of SCR KIR and iKIR treated mice infected with the MSSA Newman strains at day 3 post infection as determined by CFU quantification. C) MRSA growth curve as determined via OD600 of bacteria cultured in either 10 μM of iKIR or SCR KIR in the indicated time points. Data represent the mean ± SEM from 3–4 mice from 2–3 independent experiments. *p < 0.05 vs. SCR KIR treated mice. (TIF) [file ppat.1009387.s001.tif]

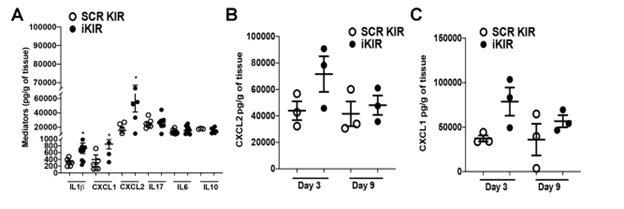

Supplement: S2 Fig — A) Detection of cytokines and chemokines in mice treated with either iKIR or SCR KIR at day 1 post-infection as measured using bead array multiplex (Eve Technologies). These results are derived from Fig 3B. B) CXCL2 and C) CXC1 abundance in skin biopsies from mice infected and treated as in A and collected at days 3 and 9 post infection. Data represent the mean ± SEM from 3–7 mice. *p < 0.05 vs. SCR KIR treated mice. (TIF) [file ppat.1009387.s002.tif]

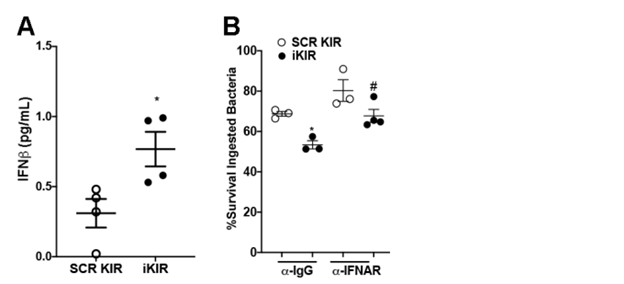

Supplement: S3 Fig — A) Detection of IFNβ in the supernatant of BMDMs from WT mice treated with the SCR KIR or iKIR peptide and co-cultured with MRSA for 24 h by ELISA. B) Determination of bacterial killing of GFP-tagged MRSA by BMDMs from WT mice treated with either SCR KIR or iKIR peptide and either an IFNAR blocking antibody or IgG control antibody as described in Phagocytosis and Killing assay—Methods. Data represent the mean ± SEM from 2 independent experiments. *p < 0.05 vs. SCR KIR treated. #p<0.05 vs. iKIR+ αIgG. (TIF) [file ppat.1009387.s003.tif]
